# Low Stability of Integrin-Binding Deficient Mutant of FGF1 Restricts Its Biological Activity

**DOI:** 10.3390/cells8080899

**Published:** 2019-08-15

**Authors:** Anna Szlachcic, Martyna Sochacka, Aleksandra Czyrek, Lukasz Opalinski, Daniel Krowarsch, Jacek Otlewski, Malgorzata Zakrzewska

**Affiliations:** 1 Department of Protein Engineering, Faculty of Biotechnology, University of Wroclaw, 50-383 Wroclaw, Poland; 2 Department of Protein Biotechnology, Faculty of Biotechnology, University of Wroclaw, 50-383 Wroclaw, Poland

**Keywords:** fibroblast growth factor 1, integrin α_v_β_3_, protein stability, proteolytic degradation, mitogenic activity, protein-protein interaction, anti-apoptotic activity

## Abstract

Fibroblast growth factor 1 (FGF1) has been shown to interact with integrin α_v_β_3_ through a specific binding site, involving Arg35 residue. The FGF1 mutant (R35E) with impaired integrin binding was found to be defective in its proliferative response, although it was still able to interact with FGF receptors (FGFR) and heparin and induce the activation of downstream signaling pathways. Here, we demonstrate that the lack of mitogenic potential of R35E mutant is directly caused by its decreased thermodynamic stability and susceptibility to proteolytic degradation. Introduction of three stabilizing mutations into R35E variant compensated the effect of destabilizing R35E mutation and restored the proliferation potential of FGF1. Moreover, the stabilized R35E variant regained both anti-apoptotic and wound healing activities, while remaining defective in binding to integrin α_v_β_3_. Our results suggest that the thermodynamic stability and resistance to degradation, rather than the interaction with integrin are required for mitogenic response of FGF1.

## 1. Introduction

Fibroblast growth factors (FGFs) form a family of signaling proteins involved in diverse cellular processes such as cell proliferation, migration, angiogenesis, embryonic and fetal development [1,2]. FGFs interact with specific transmembrane receptor tyrosine kinases (RTKs), FGF receptors (FGFRs), which include four prevalent types (FGFR1–4) and one lacking an intracellular kinase domain (FGFR5) [3]. FGF-FGFR interaction stimulates receptor dimerization and activation of receptor tyrosine kinases, initiating multiple signal transduction pathways. The complexity of FGF-induced signaling requires precise spatial and temporal regulation, as well as the control of the signal strength [4,5]. Regulatory mechanisms of FGFR signaling are not yet fully understood, nevertheless a growing number of proteins specifically affecting the FGF ligands and FGF-induced signaling cascades have been identified [2]. One of them is integrin α_v_β_3_ reported to bind directly to fibroblast growth factor 1 (FGF1) and influence its downstream signals [6,7].

Many reports point to the importance of integrin-growth factor receptors crosstalk [8,9]. Integrins are able to stimulate many types of RTKs, leading to the promotion of cancer growth and progression [10]. Accordingly, Takada and coworkers demonstrated that integrin α_v_β_3_ may affect the cellular response induced by FGF1 [6]. The integrin α_v_β_3_ binding site on FGF1 was identified as distinct from the receptor binding region and close to the heparin-binding site, with mostly positively charged residues responsible for the interaction [6]. FGF1 mutant (R35E) deficient in integrin binding exhibits impaired biological function, as shown both in vitro and in vivo [7,11]. R35E variant was found to be unable to stimulate cell proliferation and migration [7], and to suppress FGF1-dependent tumorigenesis in animal models [11].

On the other hand, even a single amino acid substitution, especially a charge-reversing mutation, as in the case of R35E in FGF1, can lead to a change in protein stability [12,13]. Thus, the lack of prolonged cellular response (mitogenesis), coupled with the unchanged affinity for FGF receptors and the ability to induce downstream signaling (short-term response typical for unstable FGFs), suggested to us that R35E FGF1 mutant may exhibit reduced stability. Since the stabilization of FGF ligands with heparan sulfates or specific mutations [14], as well as the decreasing temperature of cell culture experiments restores the ability of unstable FGFs to induce cellular response [15], we have decided to verify if protein stability plays a role in the reported effects of R35E FGF1 variant on the cellular outcome. To this end, we took advantage of previously described stabilizing mutations, Q40P, S47I, and H93G, which increase the thermodynamic stability and proteolytic resistance of FGF1, but have no impact on its binding to FGFRs or any other properties [14,16]. Here, we present a detailed analysis of integrin-binding deficient mutants of FGF1 with diverse stability and biological activities.

## 2. Materials and Methods

### 2.1. Construction, Expression and Purification of Recombinant Proteins

The coding sequence of truncated form of human FGF1 (Ala-FGF1^21–154^) cloned in the pET-3c vector was used. R35E mutation [6] and stabilizing mutations (Q40P, S47I, H93G) [14] were introduced with QuikChange™ site-directed mutagenesis kit (Stratagene, La Jolla, CA, USA) using mutagenic primers (Genomed, Warsaw, Poland). Recombinant FGF1 variants were expressed and purified as described before [17]. Since we used the truncated form of FGF1, we applied a numbering system according to Gimenez-Gallego et al. (1–140) [18]. The full length extracellular fragment of FGFR1-IIIc fused to the Fc (FGFR1c-Fc) was produced in CHO cells and purified using Protein A Sepharose (GE Healthcare, Piscataway, NJ, USA), as described previously by our group [19]. Purity and identity of protein samples were confirmed by SDS-PAGE and mass spectrometry.

### 2.2. Thermal Stability Measurements

To determine the stability of FGF1 variants, thermal denaturation curves were acquired following the changes in the ellipticity signal at 227 nm. Measurements were performed at a protein concentration 4 × 10^−6^ M in the presence of 0.7 M GdmCl in 25 mM sodium phosphate buffer, pH 7.3 in a cuvette of 10 mm path length, using a scan rate of 0.25 °C/min, as described previously [16]. Thermodynamic data were analyzed using PeakFit software (Jandel Scientific Software, San Jose, CA, USA), assuming a two-state reversible equilibrium transition.

### 2.3. Analysis of FGF1 Variants Interaction with FGFR1

Binding kinetics were measured using bio-layer interferometry (BLI) techniques with ForteBio Octet K2 (Pall ForteBio, San Jose, CA, USA). All steps of interaction studies were performed at 25 °C with shaking at 1000 rpm in PBS buffer. The 96-microwell plates were filled with 200 μL of buffer or samples and incubated for 10 min at 25 °C prior measurements for system stabilization. Protein A biosensors were hydrated in PBS buffer for 10 min and then loaded with FGFR1c-Fc (20 μg/mL for 600 s). Association and dissociation phases (180 s each) were monitored at various concentrations of FGF1 proteins ranging from 100 to 800 nM. As a control a reference sensor without FGFR1c-Fc was used. Kinetic parameters were determined by global fitting with the 2:1 “heterogeneous ligand” model and steady state analysis (averaged response values from 170 to 180 s) from three independent experiments with ForteBio Data Analysis 11.0 software (Pall ForteBio, San Jose, CA, USA).

### 2.4. Pull Down Assay

To confirm the interaction of FGF1 variants with integrin α_v_β_3_ 1 µg of His-tagged integrin α_v_β_3_ (R&D Systems, Minneapolis, MN, USA) was bound to Co^2+^-NTA resin (Thermo Fisher Scientific, Waltham, MA, USA) (50 µL) for 30 min at ambient temperature and washed three times with HBS buffer (10 mM HEPES, 150 mM NaCl, 1 mM Mn^2+^, pH 7.4). Next, FGF1 variants in HBS buffer were added at concentration of 50 ng/mL (200 µL) and incubated at 4 °C overnight. Resin was washed three times with HBS buffer and bound proteins were eluted with SDS-loading buffer. Eluates were analyzed by western blotting with anti-FGF1 antibody. The presence of equal amounts of integrin α_v_β_3_ in each sample was confirmed by Coomassie Brilliant Blue staining. As a control for unspecific FGF1 interaction, a resin sample without immobilized integrin α_v_β_3_ was used.

### 2.5. Heparin Affinity Measurements

FGF1 affinity for heparin was evaluated using a HiTrap Heparin-Sepharose column (GE Healthcare) and a linear NaCl gradient (in 25 mM Tris-HCl, pH 7.4) in a range from 0.1 M to 2 M using AKTA Explorer FPLC system (GE Healthcare).

### 2.6. Cell Culture

Mouse embryo fibroblast cells (NIH 3T3) obtained from American Type Culture Collection (ATCC, Manassas, VA, USA) were cultivated in DMEM (Thermo Fisher Scientific) supplemented with 10% fetal bovine serum (Thermo Fisher Scientific) and antibiotics (100 U/mL penicillin, 100 µg/mL streptomycin). Murine pro B cell line (BaF3) transfected with FGFR1-IIIc (BaF3-R1c), was a kind gift from Dr. David Ornitz from the Department of Developmental Biology, Washington University School of Medicine. The cells were cultured in RPMI-1640 medium (Thermo Fisher Scientific) supplemented with 10% newborn bovine calf serum (Thermo Fisher Scientific), antibiotics (100 U/mL penicillin, 100 µg/mL streptomycin), β-mercaptoethanol (50 nM) and mouse interleukin 3 (IL-3, PeproTech, Hamburg, Germany). Human osteosarcoma cell line, U2OS, stably transfected with FGFR1-IIIc (U2OS-R1) was provided by Dr. Ellen M. Haugsten from the Department of Molecular Cell Biology, Institute for Cancer Research (Oslo University Hospital). U2OS-R1 cells were grown in DMEM (Biowest) supplemented with 10% fetal bovine serum (Thermo Fisher Scientific) and antibiotics (100 U/mL penicillin, 100 µg/mL streptomycin and 1 mg/mL geneticin).

### 2.7. Analysis of Signaling Pathways

NIH 3T3 cells were serum-starved for 6 h and stimulated for 15 min with 10 ng/mL of FGF1 variants in the presence of 10 U/mL heparin (Sigma-Aldrich, St. Louis, MO, USA). Cells were then washed with PBS, lysed with SDS sample buffer and sonicated. Total cell lysates were subjected to SDS-PAGE separation and western blotting analysis with mouse anti-phospho-FGFR (Tyr653/Tyr654) antibody (p-FGFR) (#3476), mouse anti-phospho-p44/42 (Thr202/Tyr204) MAP kinase antibody (p-Erk1/2) (#9106) and rabbit anti-p44/42 MAP kinase antibody (Erk1/2) (#9102) from Cell Signaling Technology (Danvers, MA, USA), anti-phospho-PLCγ (Tyr783) antibody (sc-12943) (p-PLCγ) from Santa Cruz Biotechnology (Dallas, TX, USA). Mouse anti-Hsp90 antibody (610418) from BD Transduction Laboratory (San Jose, CA, USA) was used as a control of equal sample loading. Horseradish peroxidase-conjugated secondary antibodies from Jackson ImmunoResearch (West Grove, PA, USA) and chemiluminescent substrate (Thermo Fisher Scientific) were used for visualization in the ChemiDoc station (BioRad, Hercules, CA, USA). ImageLab software (version 6.0.1) from Biorad was used to quantify the intensities of the bands of interest. The intensity of bands corresponding to phospho-proteins (p-FGFR, p-Erk1/2, p-PLCγ) was normalized to the intensity of Hsp90 bands and then expressed as a fraction of the response observed for the wild-type FGF1.

### 2.8. Microscopy

U2OS-R1 cells were incubated with 10 µg/mL of DyLight550-labeled wild type FGF1 alone or in combination with non-labeled FGF1 R35E mutant in 1:1 and 1:10 molar ratio, for 1 h on ice. The cells were subsequently washed with PBS and fixed with 4% paraformaldehyde (20 min, at 4 °C). Next, the cells were washed with PBS and nuclei were stained for 10 min with NucBlue Live dye (Thermo Fisher Scientific). Wide-field fluorescence microscopy was carried out using a Zeiss Axio Observer Z1 microscope (Zeiss, Oberkochen, Germany). Images were taken using LD-Plan-Neofluar 40×/0.6 Korr M27 objective and Axiocam 503 camera. FGF1-DyLight550 signal was visualized with 540/552-nm bandpass excitation filter and 575/640-nm bandpass emission filter. NucBlue Live signal was visualized with 335/383-nm bandpass excitation filter and 420/470-nm emission filter. Image analysis was carried out using ZEN 2.3 (Zeiss, Oberkochen, Germany) and ImageJ (NIH, Bethesda, MD, USA).

### 2.9. FGF1-Induced Proliferative Response

24-h-starved NIH 3T3 and BaF3-R1c cells grown on the 96-well plates were treated with increasing concentrations (1, 3, 10, 30, and 100 ng/mL) of FGF1 variants in the presence of heparin (10 U/mL or 0.36 U/mL). After 48 h, cell viability was determined by addition of AlamarBlue Cell Viability Reagent (Thermo Fisher Scientific) for 4 h. The emission of fluorescent reduced form of the dye was measured at 590 nm upon excitation at 560 nm on Infinite M1000 PRO plate reader (Tecan, Männedorf, Switzerland). The data were normalized and expressed as a percentage of maximal response observed for the wild-type.

### 2.10. Protein Stability in Cell-Conditioned Medium

NIH 3T3 cells were starved in a serum-free medium for 24 h. FGF1 variants were added to the medium to a final concentration of 50 ng/mL, in the presence of 10 U/mL heparin, and incubated with cells for 48 h at 37 °C. Then, conditioned medium was aspirated and added to the new set of 24-h-starved NIH 3T3 cells. Activation of cell signaling cascades was used as a sensitive readout of FGF1 proteins degradation. Freshly prepared 50 ng/mL FGF1 solutions served as positive controls. Cells were incubated for 15 min at 37 °C and lysed with SDS sample buffer. The total cell lysate was separated by SDS-PAGE and analyzed by western blotting.

### 2.11. Limited Proteolysis with Trypsin

FGF1 variants (200 µg/mL) were subjected to the limited proteolysis with trypsin (15 µg/mL) (Worthington Biochemical Corporation, Lakewood, NJ, USA), in reaction buffer (100 mM TrisHCl, 20 mM CaCl_2_, pH 8.3) at 37 °C. Proteolysis was terminated by the addition of SDS sample buffer at different time points. Proteolytic fragments were visualized by SDS-PAGE and Coomassie Brilliant Blue staining.

For the quantitative measurement of FGF1 variants susceptibility to proteolysis, the fluorescence of tryptophan was monitored as a readout of degraded protein fraction. Proteolytic reactions were carried out with 80 µg/mL of FGF1, 6 µg/mL of trypsin in the reaction buffer at 37 °C. Fluorescence emission measurements at 353 nm upon excitation at 280 nm were performed using FP-750 spectrofluorimeter (Jasco, Easton, MD, USA). The rate of proteolysis was determined as a slope of the linear regression of tryptophane fluorescence signal versus time during the first 300 s.

### 2.12. Half-Life Analysis

There were 24-h serum starved NIH 3T3 cells treated with FGF1 variants (100 ng/mL) in the presence of 10 U/mL heparin and incubated at 37 °C. At different time points (0, 12, 24 and 48 h), medium was aspirated and then incubated with 30 μL of Heparin-Sepharose resin (GE Healthcare) for 2 h at 4 °C. In addition, FGF1 variants (500 ng/mL) were incubated at 37 °C in human serum (H4522, Sigma-Aldrich) in the presence of 10 U/mL heparin. At different time points (0, 12, 24 and 48 h), 200 μL of sample was aspirated and then incubated with 30 μL of Heparin-Sepharose resin for 2 h at 4 °C.

In each case, after washing twice with PBS, bound proteins were eluted from the resin with SDS sample buffer and analyzed by western blotting with rabbit anti-FGF1 antibody (#3139) from Cell Signaling Technology (Danvers). ImageLab software (version 6.0.1) from Biorad was used to quantify the intensities of the bands of interest and the half-life values were derived from single exponential decay equation with SigmaPlot 12 software (Systat Software).

### 2.13. Anti-Apoptotic Assay

There were 24-h serum starved NIH 3T3 cells treated for 16 h with FGF1 variants (50 ng/mL) or fetal calf serum (10%) in the presence of 0.36 U/mL or 10 U/mL heparin. Next, the caspase-3/7 activity and the cell viability were determined using ApoLive-Glo Multiplex Assay (Promega, Madison, WI, USA) according to the manufacturer’s protocol. The ratio of the caspase-3/7 activity to the cell viability was normalized towards the untreated cells, and denoted as relative caspase-3/7 activity.

### 2.14. Scratch Wound Assay

Cell migration was measured with the IncuCyte Scratch Wound Assay (Essen BioScience, Ann Arbor, MI, USA). NIH 3T3 cells seeded on a 96-well ImageLock plate were serum-starved for 24 h and scratched with WoundMaker (Essen BioScience). Then, the cells were stimulated with FGF1 variants (10 ng/mL) in the presence of heparin (10 U/mL or 0.36 U/mL) for 48 h. Every 2 h, images of the wounds were automatically acquired. The data were analyzed with respect to the spatial cell density in the wound area using the IncuCyte software package.

### 2.15. Statistical Analysis

For statistical analyses, one-way analysis of variance (ANOVA) with Tukey’s posttest was applied using SigmaPlot 12 software (Systat Software). *p* < 0.05 was considered statistically significant.

## 3. Results

### 3.1. R35E Mutation Significantly Decreases the Stability of FGF1

FGF1 R35E (R50E in the full-length numbering system) variant was reported to be defective in inducing a prolonged FGFR-dependent response, including cell proliferation and sustained downstream signaling [6,7]. This was perceived as a result of its defective integrin α_v_β_3_ binding, suggesting the importance of ternary FGF1/FGFR/integrin α_v_β_3_ complex formation for FGF1-induced signaling. Since it was previously demonstrated that thermodynamic stability strongly influences the biological activity of FGF proteins [14,15], the reported lack of mitogenic potential of FGF1 R35E variant could be also a consequence of its decreased stability.

**Figure 1:**
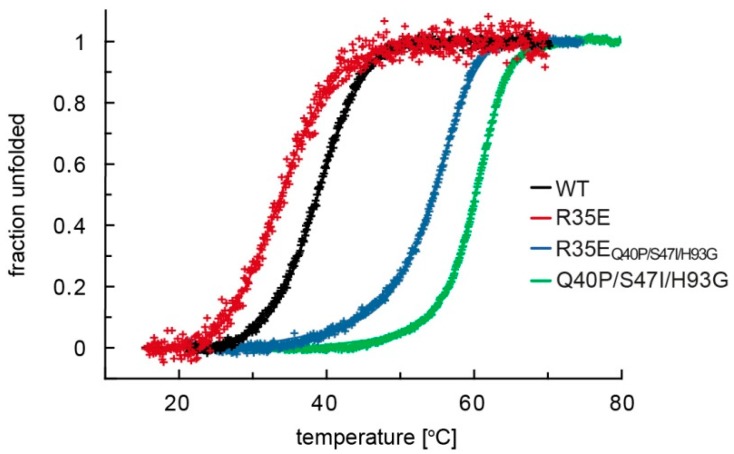
R35E mutation causes significant loss of stability of fibroblast growth factor 1 (FGF1). Normalized thermal denaturation curves of FGF1 variants monitored by the changes of ellipticity at 228 nm in the presence of 0.7 M guanidinium chloride. T_den_ of FGF1 variants were calculated assuming two-state reversible thermal transition, and equal to 39.2 °C for wild-type FGF1 (ΔH = 60.4 kcal/mol), 33.5 °C for FGF1 R35E (ΔH = 46.4 kcal/mol) and 56.2 °C for FGF1 R35E_Q40P/S47I/H93G_ (ΔH = 87.7 kcal/mol) and 60.8 °C for FGF1 Q40P/S47I/H93G (ΔH = 107.2 kcal/mol).

To check if there is a relationship between the stability of FGF1 R35E and its disturbed activity, we carried out thermodynamic analyses. We verified the stability of R35E variant by monitoring the changes in circular dichroism (CD) signal during thermal denaturation (Figure 1). Denaturation curves clearly showed that the stability of R35E protein is decreased, as T_den_ of the mutant equals to 33.5 °C and is 5.7 °C lower than T_den_ of the wild-type FGF1 (39.2 °C) (Figure 1).

### 3.2. Compensation Effect of Stabilizing Substitutions in Integrin-Binding Deficient Variant

Having at our disposal mutants of FGF1 with significantly improved stability, we generated a variant of FGF1 that combined R35E mutation and three stabilizing substitutions described before, Q40P, S47I, and H93G, which increase the denaturation temperature of FGF1 to 60.8 °C (Figure 1) [17]. In principle, such a variant should still be unable to bind integrin α_v_β_3_, compensating at the same time the R35E mutation-accompanied loss of stability. This allowed us to study the role of FGF1-integrin binding independently from the consequences of low protein stability. Indeed, R35E/Q40P/S47I/H93G mutant, denoted as R35E_Q40P/S47I/H93G_ showed increased thermal stability reflected in the significantly higher T_den_ (56.2 °C), as compared to the T_den_ of R35E variant (33.5 °C) (Figure 1).

### 3.3. Analysis of the Effect of Mutations on FGF1 Binding Properties

**Figure 2:**
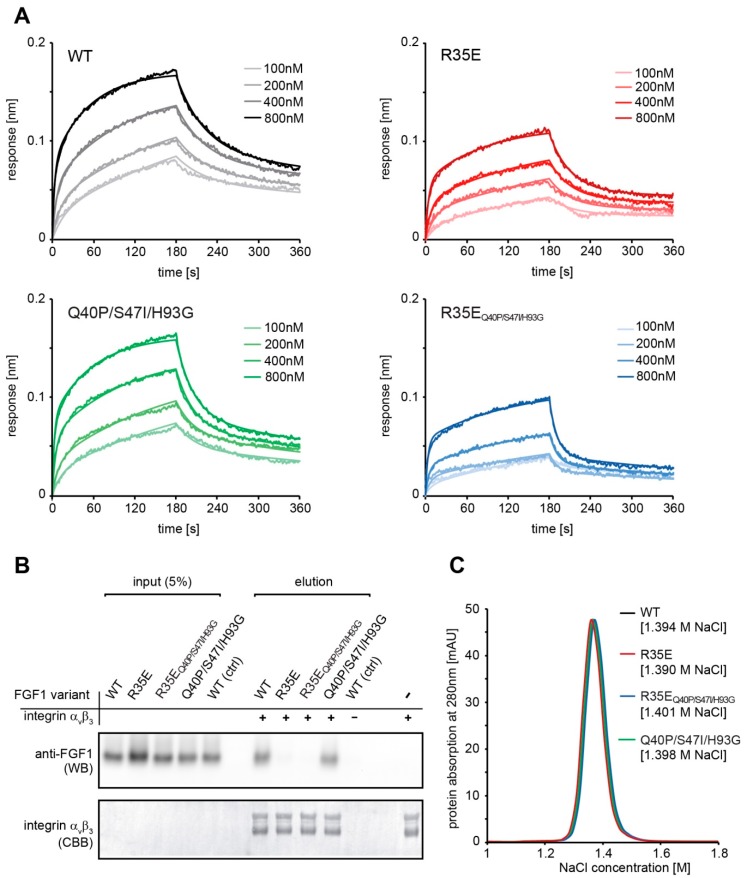
Stabilizing mutations do not interfere with binding properties of FGF1. (**A**) Binding profiles of FGF1 variants to FGFR1 were measured by the bio-layer interferometry (BLI) technique. Serial twofold dilutions of the FGF1 variants were analyzed for interaction with FGFR1-Fc immobilized on sensors coated with Protein A. (**B**) Verification of FGF1 variants interaction with integrin α_v_β_3_ by pull down. Integrin α_v_β_3_ was immobilized on Co^2+^-NTA resin and incubated with FGF1 variants. FGF1 proteins bound to integrin were eluted and detected with western blotting using anti-FGF1 antibody. An equal amount of immobilized integrin α_v_β_3_ was confirmed by CBB staining. (**C**) Elution profiles of FGF1 variants from a Heparin-Sepharose column with a NaCl gradient.

In order to validate that FGF1 variants are functional in terms of receptor binding, we tested their affinity for FGFR1 using BLI measurements. Both R35E and R35E_Q40P/S47I/H93G_ variants showed only slightly reduced binding affinities for the extracellular domain of FGFR1 (FGFR1-IIIc) in BLI experiments, as compared to the wild-type FGF1 (Figure 2A). Kinetic measurements followed by global fitting with the 2:1 ligand model revealed K_D1_ and K_D2_ values equal to 4.69 × 10^−8^ M and 7.56 × 10^−8^ M for wild-type FGF1 (K_D_ (steady state analysis) = 2.70 × 10^−7^), 2.91 × 10^−7^ M and 1.52 × 10^−7^ M for R35E (K_D_ (steady state analysis) = 4.60 × 10^−7^), 4.77 × 10^−7^ M and 6.90 × 10^−7^ M for R35E_Q40P/S47I/H93G_ (K_D_ (steady state analysis) = 7.20 × 10^−7^), and 1.02 × 10^−7^ and 1.05 × 10^−7^ for Q40P/S47I/H93G variant (K_D_ (steady state analysis) = 1.62 × 10^−7^), which is in agreement with data reported before for the FGF1-FGFR1 interaction [20,21].

Since even a single substitution can strongly influence protein-protein interaction, we also verified if stabilizing mutations did not interfere with integrin-binding properties of FGF1. Using a pull-down technique, we showed the inability to bind integrin α_v_β_3_ for both R35E and its stabilized version (R35E_Q40P/S47I/H93G_) (Figure 2B). At the same time, both wild-type FGF1 and a variant containing stabilizing mutations (Q40P/S47I/H93G) effectively bound to immobilized integrin α_v_β_3_ (Figure 2B). Since FGF1 R35E_Q40P/S47I/H93G_ did not interact with integrin α_v_β_3_, we confirmed that the loss of affinity of R35E mutant for integrin is not caused by the lowered stability, but is a direct consequence of the introduced point mutation.

As FGF1 binding to heparin is based on electrostatic interactions generated by positively charged arginine and lysine residues, we checked whether the affinity of FGF1 to heparin is not altered by the presence of R35E substitution or stabilizing mutations. To this end, FGF1 variants were bound to the Heparin-Sepharose column and eluted with linear NaCl gradient. The concentration of NaCl, required for protein elution, reflected FGF1 affinity for heparin. All studied FGF1 proteins were eluted at a virtually identical salt concentration, indicating the unchanged affinity of FGF1 mutants for heparin (Figure 2C).

### 3.4. Stabilizing Mutations Restore Biological Competence of R35E Variant

**Figure 3:**
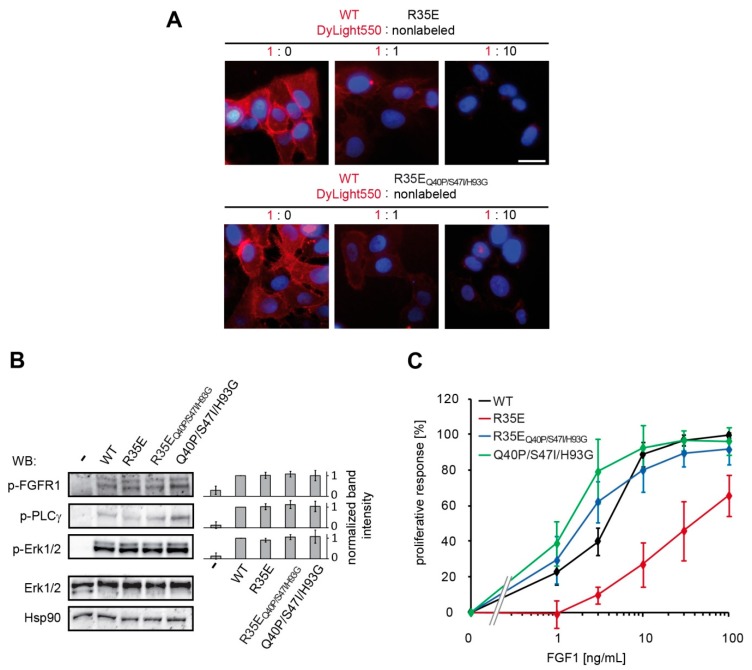
Biological activity of R35E FGF1 mutants. (**A**) U2OS-R1 cells were incubated with DyLight550-labeled wild type FGF1 alone or in combination with non-labeled R35E mutants in 1:1 and 1:10 molar ratio. Nuclei were visualized with NucLive Blue dye. (**B**) Serum-starved NIH 3T3 cells were stimulated with 10 ng/mL FGF1 variants in the presence of heparin (10 U/mL) for 15 min and the activation of downstream signaling cascades was detected with immunoblotting using the following antibodies: anti-phospho-FGFR (Tyr653/Tyr654) (p-FGFR), anti-phospho-PLC-γ (Tyr 783) (p-PLCγ), anti-phospho-Erk1/2 (p-Erk1/2), anti-Erk1/2 and anti-Hsp90 as a loading control. Representative experiment is shown, *n* = 3. The bars present quantification of bands corresponding to phospho-proteins (p-FGFR1, p-Erk1/2, p-PLCγ) normalized to loading control (Hsp90) and expressed as a fraction of the wild-type response. Data are means ± SD of three independent experiments. (**C**) 24-h serum-starved NIH 3T3 cells were treated with FGF1 variants at various concentrations (1–100 ng/mL) in the presence of heparin (10 U/mL). After 48 h viable cells was quantified using AlamarBlue reagent. Proliferative effect was normalized to the maximum response of wild-type FGF1. The data shown are mean values of five independent experiments ± SD.

In the next step, we studied the biological activity of R35E variants in terms of short-term response. To confirm the data obtained in BLI measurements using recombinant FGFR1, we performed the binding experiments in U2OS-R1 cells. We found that both R35E mutants (the single mutant and the stabilized variant) efficiently bound FGF receptors on the cell surface, as they could outcompete binding of fluorescently-labeled wild-type FGF1 in a concentration-dependent manner (Figure 3A, Figure S1A). No difference in the fluorescence intensity, and thus in the binding to FGFRs, between R35E mutants was observed. When the cells were incubated with labeled wild type FGF1 and non-labeled R35E mutants in 1:1 ratio, the average signal intensity was slightly below the half of the signal detected for the wild-type alone (Figure S1A). Next, we checked whether the binding of both R35E variants to the cellular pool of FGFRs resulted in their activation. To this end, we analyzed FGF-induced signaling pathways in NIH 3T3 upon 15-min stimulation with FGF1 proteins. We observed no significant differences in the phosphorylation of FGFR, PLCγ and Erk1/2 induced by R35E variants in relation to the wild-type FGF1 and Q40P/S47I/H93G variant. This indicates that both integrin-deficient mutants are fully competent in FGFR activation and evoking short-term cellular response (Figure 3B).

However, when we looked at the proliferative activity, which required a prolonged exposure of cells to the growth factor, we found, in agreement with previously published data [6,7], that R35E variant is defective in stimulating mitogenic response at concentrations ranging from 0.1 ng/mL to 100 ng/mL, (Figure 3C). Interestingly, the stabilization of R35E with Q40P, S47I and H93G mutations rescued the proliferative potential, suggesting that its loss of activity resulted from the impaired stability, but not its inability to bind integrin α_v_β_3_. Since heparin is a known stabilizing agent for FGF1 strongly influencing the mitogenic activity of wild-type FGF1 [14], we decided to test if the lower concentration of heparin, 0.36 U/mL [6,7] would alter the mitogenic activity of FGF1 variants. Lowered heparin concentration had a minor effect on FGF1 R35E-induced proliferation of NIH 3T3 cells, and caused virtually no change in the case of FGF1 WT and FGF1 R35E_Q40P/S47I/H93G_ (Figure S1A). However, as NIH 3T3 cells possess heparan sulfate glycosaminoglycans (HSGAGs) on their surface, the stabilizing effect of the exogenously added heparin cannot be completely separated from that of HSGAGs. Therefore, we performed additional proliferation assays in BaF3 cells expressing no HSGAGs and transfected with FGFR1c (BaF3-R1c) [22]. In this system, R35E mutant fully lost its proliferative activity, with negligible stimulation of BaF3-R1c cells even at high concentrations as 100 ng/mL (Figure S1B). These results further indicate R35E mutant’s instability, as a main driving force for its long-term activity loss.

### 3.5. R35E Mutation Increases the Susceptibility of FGF1 to Degradation

To further investigate how protein stability influences the mitogenic potential, we examined the biological activity of FGF1 variants upon the incubation with cells for the time of mitogenic assay. We incubated wild-type FGF1, R35E and R35E_Q40P/S47I/H93G_ in the presence of heparin (10 U/mL) with NIH 3T3 cells for 48 h, and then aspirated cell-conditioned media. Activation of FGF1-induced signaling cascades was used as a sensitive read-out of quantities of FGF1 variants that remained functional in media. A new set of NIH 3T3 cells was stimulated either with freshly prepared FGF1 and its variants in the presence of heparin (10 U/mL) or conditioned media after 48-h incubation, and the activation of FGFR and downstream kinases was evaluated by western blotting.

**Figure 4:**
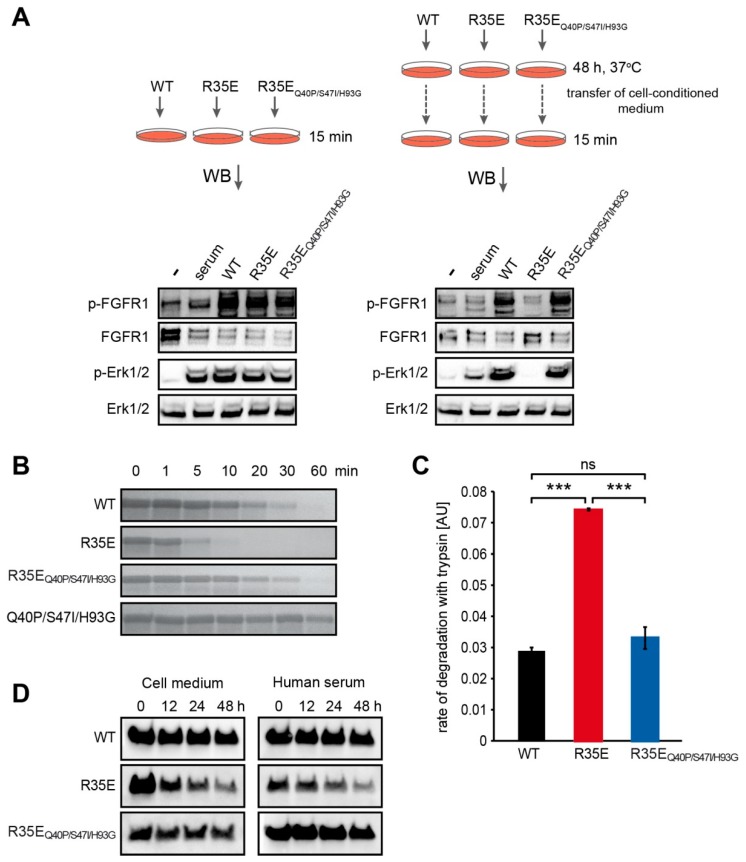
Decreased stability of R35E mutant. (**A**) Stability of FGF1 variants upon incubation with NIH 3T3 cells. Serum-starved NIH 3T3 cells were stimulated for 15 min with either freshly prepared 50 ng/mL of FGF1 variants and 10 U/mL heparin (left panel) or cell-conditioned media after 48-h incubation with 50 ng/mL FGF1 variants in the presence of 10 U/mL heparin (right panel). Activation of FGFR downstream signaling was evaluated by immunoblotting. (**B**,**C**) Limited proteolysis of FGF1 variants using trypsin. (**B**) FGF1 proteins (200 µg/mL) were treated with trypsin (15 µg/mL) and their degradation was monitored by SDS-PAGE. (**C**) Rate of proteolytic degradation of FGF1 variants (200 µg/mL) by trypsin (15 µg/mL) determined based on fluorescence emission intensity at 353 nm upon excitation at 280 nm. Slope of the linear regression of tryptophan fluorescence signal as a function of time was used as a measure of FGF1 proteolytic susceptibility. The data shown are mean values of three independent experiments ± SD. Statistical significance: ns—not significant, *** *p* < 0.001. (**D**) Degradation of FGF1 in NIH 3T3 cell-conditioned medium and in human serum. Serum-starved NIH 3T3 cells or human serum were incubated in the presence of heparin (10 U/mL) with 100 ng/mL or 500 ng/mL of FGF1 variants, respectively. The intact FGF1 proteins were recovered by affinity purification and detected by immunoblotting using anti-FGF1 antibody.

As shown on Figure 4A, there was a dramatic decrease in the level of both FGFR and Erk1/2 phosphorylation in response to R35E mutant after 48-h incubation with cells. Such change was not observed neither in the case of wild-type FGF1 nor R35E_Q40P/S47I/H93G_ variant, which evoked similar activation of FGFR receptor, regardless if they had been incubated with cells or not. This indicated that the amount of active R35E FGF1 was strongly reduced by the incubation process, whereas the concentration of functional R35E_Q40P/S47I/H93G_ was not altered. We assume that the observed effect is caused by the unfolding and/or degradation of R35E variant in the medium due to its low stability.

It is possible that the loss of FGF1 activity in cell-conditioned medium is a direct consequence of proteolytic degradation by cell-secreted proteases. Therefore, we performed the limited proteolysis of FGF1 and its mutants with trypsin. Visualizing the reaction progress in SDS-PAGE, we observed a clear difference in the susceptibility of FGF1 R35E to proteolysis that was degraded significantly faster than wild-type FGF1 and R35E_Q40P/S47I/H93G_ variant (Figure 4B). We also monitored the rate of protein degradation using tryptophan fluorescence emission change, as a measure of FGF1 proteolytic degradation (Figure 4C, Figure S2A). In the case of the intact, properly folded FGF1 protein upon excitation at 280 nm the tryptophan fluorescence emission is almost completely quenched. In the course of proteolysis, Trp107 is getting exposed and strong emission at 353 nm is observed. The slope of linear regression of tryptophan fluorescence signal as a function of time differed considerably between wild-type FGF1 and R35E variant treated with trypsin. R35E_Q40P/S47I/H93G_ variant showed virtually the same proteolytic stability as the wild-type FGF1, which corresponds accurately to the progress of degradation detected by SDS-PAGE. The susceptibility to proteolysis of R35E variant nicely correlates with its low denaturation temperature determined in the thermodynamic stability assessment (Figure 1).

Next, we analyzed directly the half-life of FGF1 variants in cell medium and human serum in the presence of heparin at the concentration used in proliferation experiment (10 U/mL) (Figure 4D). We observed that R35E mutant disappeared from the medium much faster than the wild-type, exhibiting half-life more than 10 times shorter (Figure S2B). The half-life of R35E was approximately 8 h, whereas the half-life of the wild-type and R35E_Q40P/S47I/H93G_ variant was over 100 h. Similar effect was found when FGF1 variants were incubated with human serum. The wild-type FGF1 and R35E_Q40P/S47I/H93G_ variant exhibited virtually the same half-life (~200 h), which was more than five times longer than the half-life of R35E mutant (~38 h) (Figure 4D, Figure S2B). Overall, our results indicate that the lack of stability of R35E variant leads to its degradation during 48-h incubation with cells at 37 °C via thermal unfolding and extracellular protease digestion or aggregation. This effect can be effectively reversed by stabilizing the protein structure with three point substitutions.

### 3.6. Excess of Heparin Can Partially Restore Biological Activity of R35E Mutant

Besides the mitogenic effect, FGF1 plays an important role in cell protection against apoptosis and stimulation of cell migration [23,24]. Thus, we analyzed the effect of R35E substitution in wound healing and cell apoptosis assays. First we verified anti-apoptotic properties of FGF1 mutants in serum-starved NIH 3T3 cells by measuring the caspase-3/7 activity and the cell viability.

**Figure 5:**
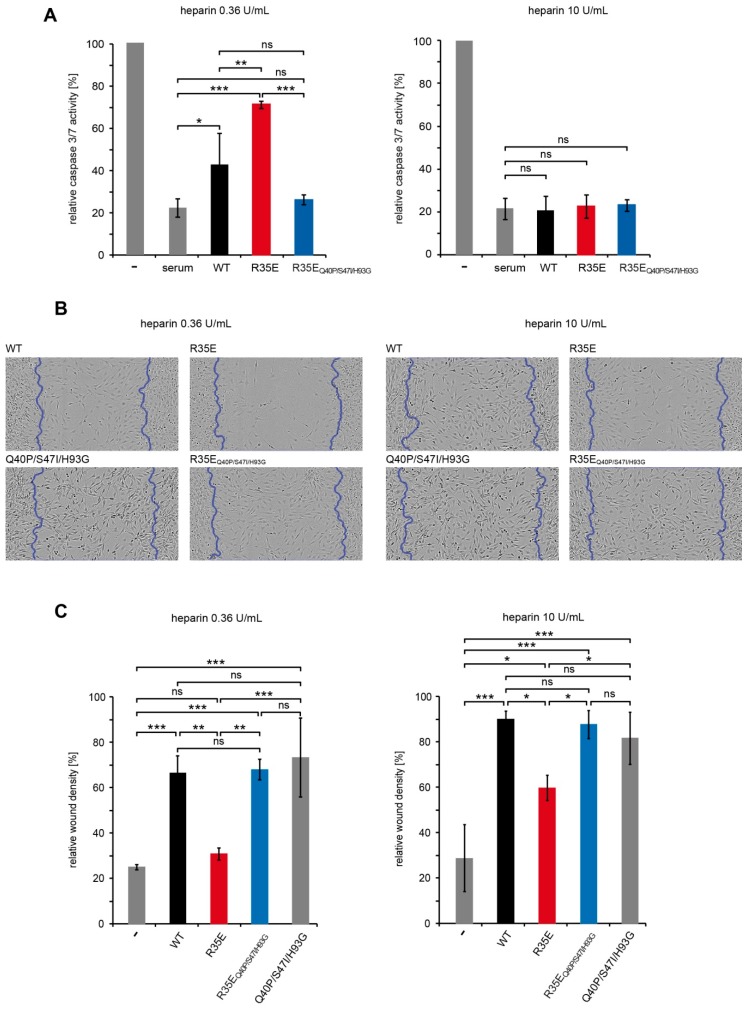
Biological activity of R35E variant depends on heparin concentration. (**A**) The anti-apoptotic activity of R35E variant in the presence of 0.36 U/mL and 10 U/mL of heparin. NIH 3T3 cells starved for 24 h were treated with bovine calf serum or FGF1 variants and the caspase-3/7 activity and cell viability were determined using ApoLive-Glo Multiplex Assay. Apoptosis levels were calculated as the ratio of caspase-3/7 activity to cell viability and normalized towards untreated cells (relative caspase-3/7 activity). The data shown are mean values of three (*n* = 3) independent experiments ± SD. Statistical significance: ns—not significant, * *p* < 0.05, ** *p* < 0.01, *** *p* < 0.001. (**B**,**C**) Effect of FGF1 variants on migration of NIH 3T3 cells. Serum-starved NIH 3T3 cells were treated with FGF1 variants (10 ng/mL) in the presence of heparin (0.36 or 10 U/mL) for 36 h. (**B**) Images of the wounds were obtained by IncuCyte Zoom software (**C**) and the relative wound density was calculated. The data shown are mean values of three (*n* = 3) independent experiments ± SD. Statistical significance: ns—not significant, * *p* < 0.05, ** *p* < 0.01, *** *p* < 0.001.

The protective effects of tested FGF1 variants differed significantly and the results varied depending on heparin concentration (Figure 5A). For the lower heparin concentration (0.36 U/mL), used previously by others [7,11], we found a reduced anti-apoptotic effect of R35E mutant, as compared to the wild-type. This observation is in agreement with the results presented above, since relatively long incubation time (16 h) with cells at 37 °C may lead to the degradation or aggregation of unstable R35E variant, but not its stabilized version (R35E_Q40P/S47I/H93G_). Interestingly, when high heparin concentration was used (10 U/mL), there was no significant difference in the anti-apoptotic activity of all tested FGF1 proteins (Figure 5A).

In the presence of low concentrations of heparin R35E variant showed a significantly reduced potential for induction of cell migration [6]. Again, this effect could be overcome by the introduction of stabilizing mutations or, to some extent, by high heparin concentration (10 U/mL) as tested in in vitro wound healing experiments (Figure 2B,C). Interestingly, stabilized R35E variant (R35E_Q40P/S47I/H93G_) stimulated cell migration as the wild-type FGF1 and Q40P/S47I/H93G, independently of heparin concentration (Figure 2B,C).

## 4. Discussion

The R35E (R50E in full-length numbering system) variant of FGF1 designed and described by Takada and colleagues is unable to stimulate cell proliferation, even though it initiates FGFR-dependent signaling [6]. This mutant was found to be defective in integrin α_v_β_3_ binding and therefore FGF1-integrin interaction was considered to be critical for FGF1 mitogenic response [6,7].

We reasoned that the observed lack of long-term cellular response to R35E mutant, together with its full functionality for the short period of time reflected in the FGF receptor activation, could be a result of decreased half-life of the protein. To verify this hypothesis, we determined the thermal stability of this mutant (Figure 1) and its susceptibility to degradation (Figure 4). We found that R35E variant is significantly less stable than the wild-type FGF1, with denaturation temperature lowered by almost 6 degrees. The thermodynamic instability promoted FGF1 degradation and reduced half-life, which prevents a long-term response, such as cell proliferation. This is in agreement with previous observation that a maximal mitogenic activity requires the presence of active FGF1 for over 12 h [25,26]. Further, we observed a strong correlation between the stability/proteolytic degradation of FGF1 mutants and their biological activity [14,17]. Accordingly, here we showed that the increase in the thermal stability of R35E mutant by the introduction of three well characterized stabilizing mutations (Q40P, S47I, H93G) significantly reduced its susceptibility to proteolysis by trypsin and prolonged its half-life (Figure 4, Figure S2), resulting in restoration of its mitogenic potential in NIH 3T3 as well as BaF3-R1c cells (Figure 3, Figure S1). We also observed the activity rescue for the stabilized variant R35E_Q40P/S47I/H93G_ in the migration assay and in the anti-apoptotic response (Figure 5). Thus, these results indicate that the low stability, rather than the defective integrin binding, is a major cause for the lack of mitogenic activity of R35E mutant.

The same effects were observed in the case of STAT3 in autosomal dominant hyper-IgE syndrome, where destabilizing mutations decreased the half-life of the protein leading to its dysfunction [27]. Similarly, loss of function of tumor suppressor, merlin, causing neurofibromatosis type 2, was directly correlated with a reduction in protein half-life and its increased degradation. Moreover, it was shown that merlin mutants maintain intrinsic functional capacity [28]. In the same manner, despite the low stability, FGF1 R35E variant was able to evoke short-term response, such as endocytosis, FGFR activation and initiation of downstream signaling. However, the incubation with cells, due to an elevated temperature and extracellular proteases’ action, deactivated the FGF1 R35E variant, while not affecting its stabilized version (R35E_Q40P/S47I/H93G_) (Figure 4).

Importantly, none of the stabilizing mutations affected the interaction of FGF1 with integrin α_v_β_3_. The stabilized variant, R35E_Q40P/S47I/H93G_, similarly to single R35E mutant, exhibit no affinity for recombinant integrin α_v_β_3_. We also verified the ability of these two mutants to bind FGFR1 using BLI technique (Figure 2). The slight decrease in the receptor affinity observed for the FGF1 variants containing R35E mutation can be explained by the involvement of Arg35 residue in the FGF1-FGFR1 interaction, as seen in the crystal structures of FGF1-FGFR1 complex [29]. Side-chain of Arg35 forms van der Waals contacts with carbonyl carbon and Cβ of Glu162 of D2 domain of FGFR1.

Since Arg35 residue is positively charged and could participate in the FGF1 interaction with negatively charged heparin or cell-surface heparans, which strongly stabilize the FGF1-FGFR complex and protect FGF1 molecule against degradation [26], we evaluated the affinity of R35E variant for heparin. We showed that the substitution to Glu did not change the elution profile of FGF1 from heparin column (Figure 2). However, the presence of sugar moieties on the cell surface affected the proliferative potential of the R35E mutant, as can be seen in the comparative experiment in BaF3-R1c cells negative for heparan sulfate glycosaminoglycans [22,30] and NIH 3T3 fibroblasts expressing notable level of HSGAGs. At the low heparin concentration (0.36 U/mL, used previously in R35E variant studies [6,7]) in NIH 3T3 cells R35E variant exhibited some mitogenic activity, whereas in BaF3-R1c cells it did not evoke any proliferative response (Figure S1). This discrepancy suggests that R35E variant can be stabilized and protected against the degradation by the surface heparans to certain extent, partially preserving its activity. Notably, we did not observe any difference in the mitogenic potential of the stabilized R35E_Q40P/S47I/H93G_ mutant in these two cell lines. We further confirmed that high concentration of heparin may protect the low stable R35E protein, similarly to structural changes causes by stabilizing mutations (Figure 5). In wound healing experiments we observed higher migration-inducing activity of R35E variant at 10 U/mL heparin concentration than at 0.36 U/mL. Similarly, at low heparin concentration we found significant differences in the caspase activity in serum-starved cells between R35E variant and the wild-type, but when heparin was present at high concentration all tested variants exhibited the same anti-apoptotic properties. Again the stable R35E_Q40P/S47I/H93G_ variant was similarly active independently of heparin concentration. Thus, we showed that the protective effect of heparin can be substituted by the increase in the protein stability [14].

Taking together, we have provided the explanation for the lack of mitogenic activity of R35E variant and suggested that the protein stability, rather than the integrin binding, is a driving force for the long-term response of FGF1. In light of our data the biological significance of the interaction between FGF/FGFR signaling system and integrins requires further in depth studies. We are convinced that our findings may facilitate the understanding how the stability of FGF proteins dictates specificity and duration of cellular response.

## Supplementary Materials

The following are available online at https://www.mdpi.com/2073-4409/8/8/899/s1, Figure S1. Biological activity of R35E FGF1 mutants. (A) Quantification of cell binding experiment with FGF1 variants presented in Figure 3A. The fluorescence intensity of the wild-type FGF1-DyLight550 in single cells was measured with Zen2.3 software. At least fifteen cells were quantified for each FGF1 variant. Spots represent fluorescence intensities of random individual cells. Black squares represent average fluorescence intensity. (B,C) R35E FGF1 variant can be partially stabilized by the cell surface heparan sulfate glycosaminoglycans. Serum-starved (B) NIH 3T3 cells and (C) BaF3-R1c cells were treated with FGF1 variants at various concentrations (1–100 ng/mL) in the presence of low heparin concentrations (0.36 U/mL). After 48 h viable cells was quantified using AlamarBlue reagent. Proliferative effect was normalized to the maximum response of wild-type FGF1. The data shown are mean values of three independent experiments ± SD. Figure S2. Degradation of R35E mutant. (A) Quantitative comparison of susceptibility of FGF1 variants to proteolysis. Proteolytic degradation of FGF1 variants (200 µg/mL) by trypsin (15 µg/mL) was determined based on fluorescence emission intensity at 353 nm upon excitation at 280 nm. (B) Analysis of half-lives of FGF1 variants in NIH 3T3 cell medium and human serum from western blotting experiments presented in Figure 4D. The intensities of the bands were fitted with single exponential decay equation and the half-lives were determined.
