# Supplementary material for: Low Stability of Integrin-Binding Deficient Mutant of FGF1 Restricts Its Biological Activity
Source: Cells. 2019 Aug 15;8(8):899. doi: 10.3390/cells8080899 (PMC6721657; doi:10.3390/cells8080899)
Supplement: Supplementary file 1 [file cells-08-00899-s001.pdf]

Supplementary Materials

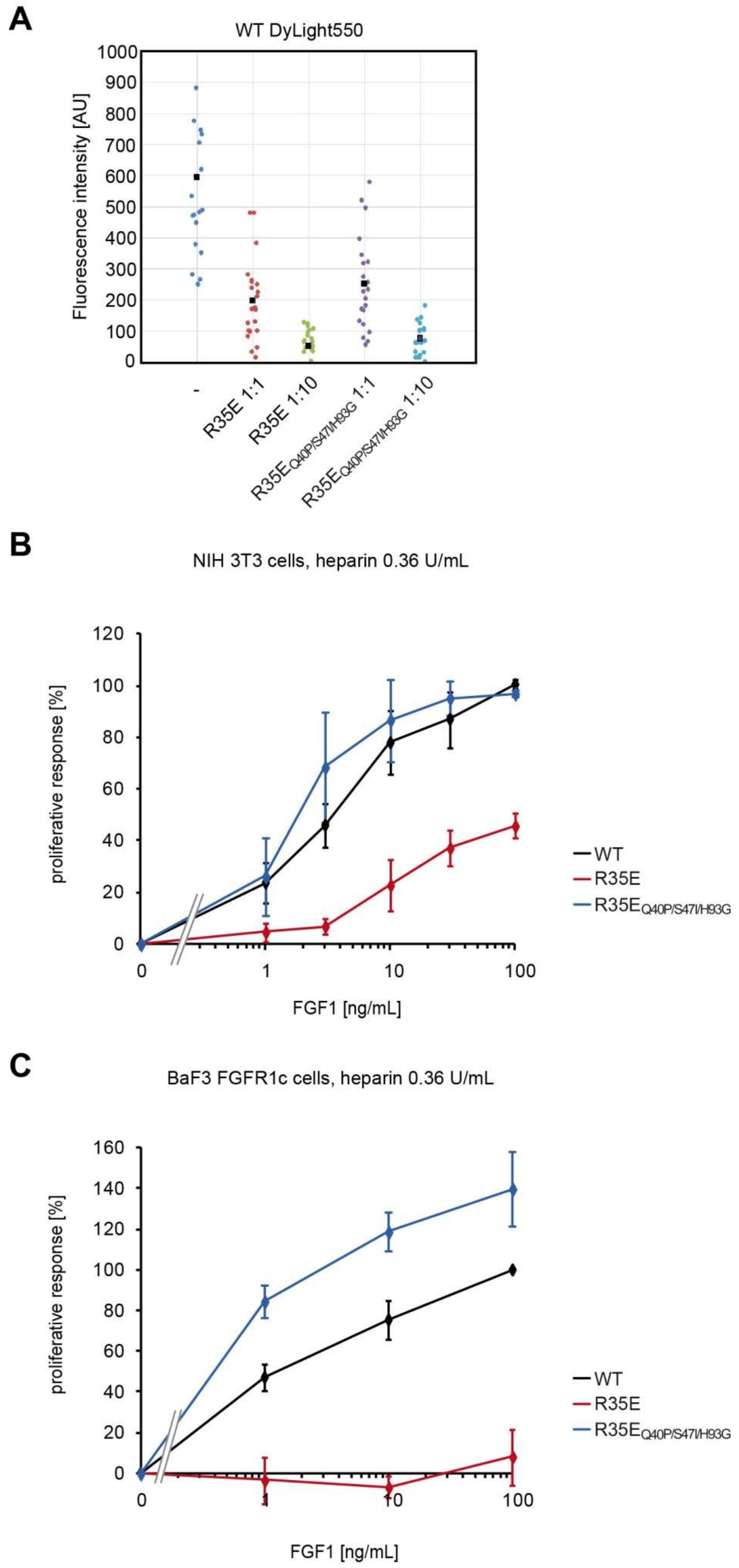

Figure S1. Biological activity of R35E FGF1 mutants. (**A**) Quantification of cell binding experiment with FGF1 variants presented in Fig. 3A. The fluorescence intensity of the wild-type FGF1-DyLight550 in single cells was measured with Zen2.3 software. At least fifteen cells were quantified for each FGF1

variant. Spots represent fluorescence intensities of random individual cells. Black squares represent average fluorescence intensity. (**B, C**) R35E FGF1 variant can be partially stabilized by the cell surface heparan sulfate glycosaminoglycans. Serum-starved (**B**) NIH 3T3 cells and (**C**) BaF3-R1c cells were treated with FGF1 variants at various concentrations (1–100 ng/mL) in the presence of low heparin concentrations (0.36 U/mL). After 48 h viable cells was quantified using AlamarBlue reagent. Proliferative effect was normalized to the maximum response of the wild-type FGF1. The data shown are mean values of three independent experiments ± SD.

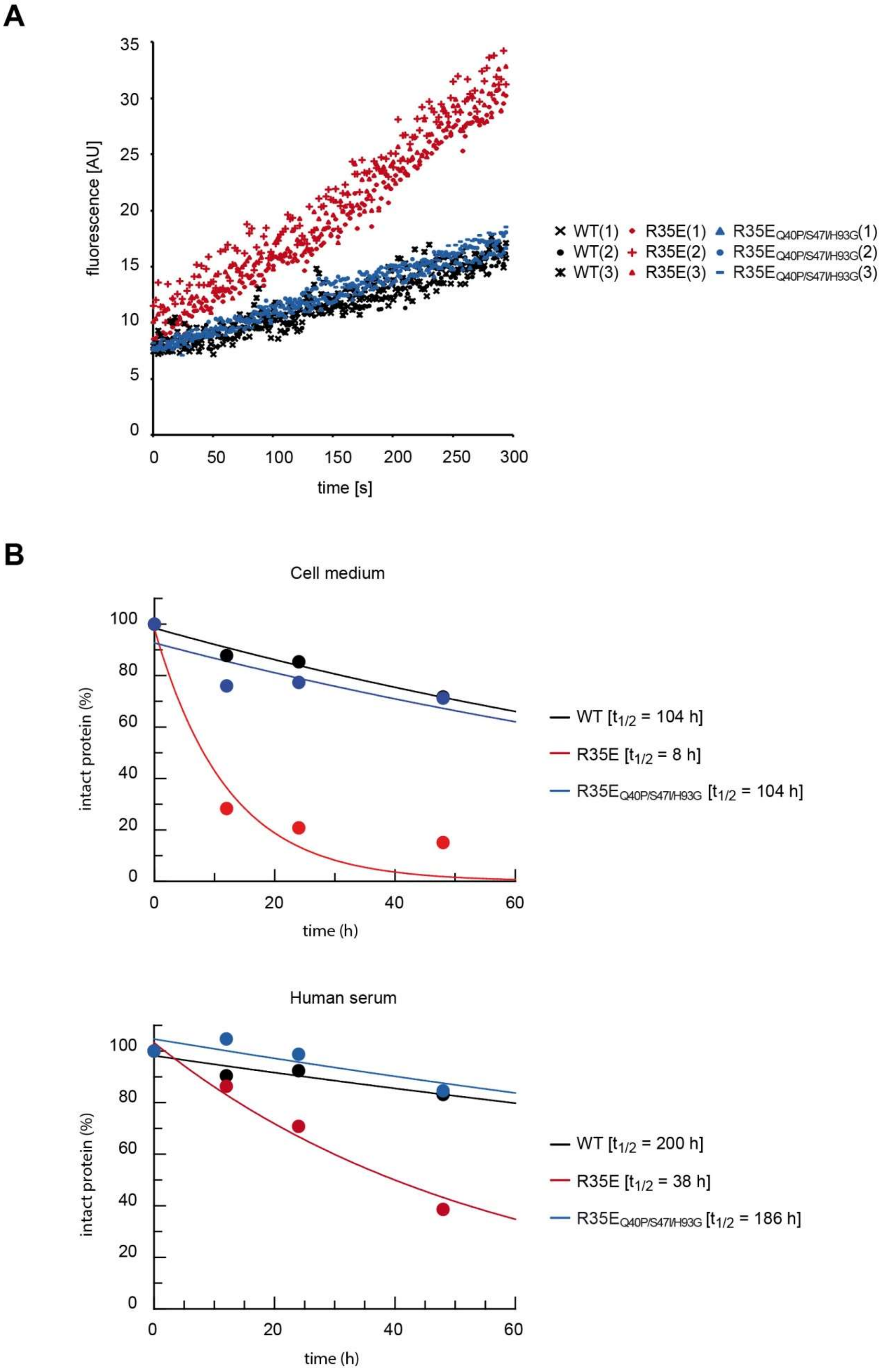

Figure S2. Degradation of R35E mutant. (**A**) Quantitative comparison of susceptibility of FGF1 variants to proteolysis. Proteolytic degradation of FGF1 variants (200 µg/mL) by trypsin (15 µg/mL) was determined based on fluorescence emission intensity at 353 nm upon excitation at 280 nm. (**B**) Analysis of half-lives of FGF1 variants in NIH 3T3 cell medium and human serum from western blotting experiments presented in Fig. 4D. The intensities of the bands were fitted with single exponential decay equation and the half-lives were determined.
